# Magnetic resonance imaging of pectoralis major injuries: a radiologist’s essential guide

**DOI:** 10.1007/s10140-025-02370-1

**Published:** 2025-08-07

**Authors:** MeNore G. Lake, Matt R. Skalski, Michael K. Chiu, Nicholas A. Lewis, Dani Sarohia, Eric A. White, Dakshesh B. Patel

**Affiliations:** 1 Musculoskeletal Radiology Division, Department of Radiology, Keck School of Medicine, University of Southern California, Los Angeles, CA USA; 2 Skalski Chiropractic Radiology, Chippewa Falls, WI USA; 3 Musculoskeletal Radiology Division, Department of Radiology, Los Angeles General Medical Center, Los Angeles, CA USA

**Keywords:** Pectoralis major, Pectoralis major injuries, Anatomy, Magnetic resonance imaging

## Abstract

The complex and variable anatomy of the pectoralis major, and the evolving description of this anatomy and its patterns of injury in the literature contribute to the challenge in applying a standardized classification scheme for injuries [1, 2]. While pectoralis major injuries are uncommon injuries, their occurrence is increasing. Many patients after this injury will present to the emergency department. It is important for emergency radiologists to understand the imaging findings of these injuries. Diagnosis with magnetic resonance imaging (MRI) can avoid surgical delay and improve patient outcomes [3]. This article presents a focused anatomic and imaging discussion of pectoralis major injuries as seen on MRI.

## Introduction

The incidence of injuries of the pectoralis major muscle has increased by up to 40% over the past two decades, highlighting the importance of radiologists’ understanding and reporting of the essential imaging features [4]. Many patients after this injury will present to the emergency department. It is important for emergency radiologists to understand the imaging findings of these injuries. Diagnosis with MRI, obtained based on clinical presentation, can avoid surgical delay and improve patient outcomes [3].

While injuries of the pectoralis major may be apparent on physical examination, including with ecchymosis which can persist for approximately six weeks, and with possible thinning of the anterior axillary fold, imaging can localize the site of injury and contribute to clinical decision-making regarding non-operative versus operative management [5–7]. The decision to obtain MRI in the emergency department will depend on local preference, availability of MRI in light of triaging acute imaging needs, and the relative time of presentation following injury. MRI may be obtained in the emergency setting for confirmation and classification of injury, but if the presentation is not relatively acute, MRI may be obtained in the outpatient setting. In the emergency department, orthopedic consultation may be of value for a more detailed clinical assessment to triage when and which of these patients may benefit from imaging to guide management.

The complex and variable anatomy and the evolving description of this anatomy in the literature contribute to the challenge in applying a standardized classification scheme for injuries [1, 2]. This article presents a focused anatomic and imaging discussion of pectoralis major injuries as seen on magnetic resonance imaging.

## Anatomy of the pectoralis major and regional structures

### Pectoralis major muscle: anatomy and function

The pectoralis major muscle is a multipennate muscle, referring to its feather-like structure, and consists of multiple variably oriented segments of variable lengths [8, 9]. It is this variation in the lengths and orientations of the constituent fibers that results in transmission of differing degrees of stress during contraction, predisposing the more inferior sternal fibers to tear [10–14].

**Fig. 1:**
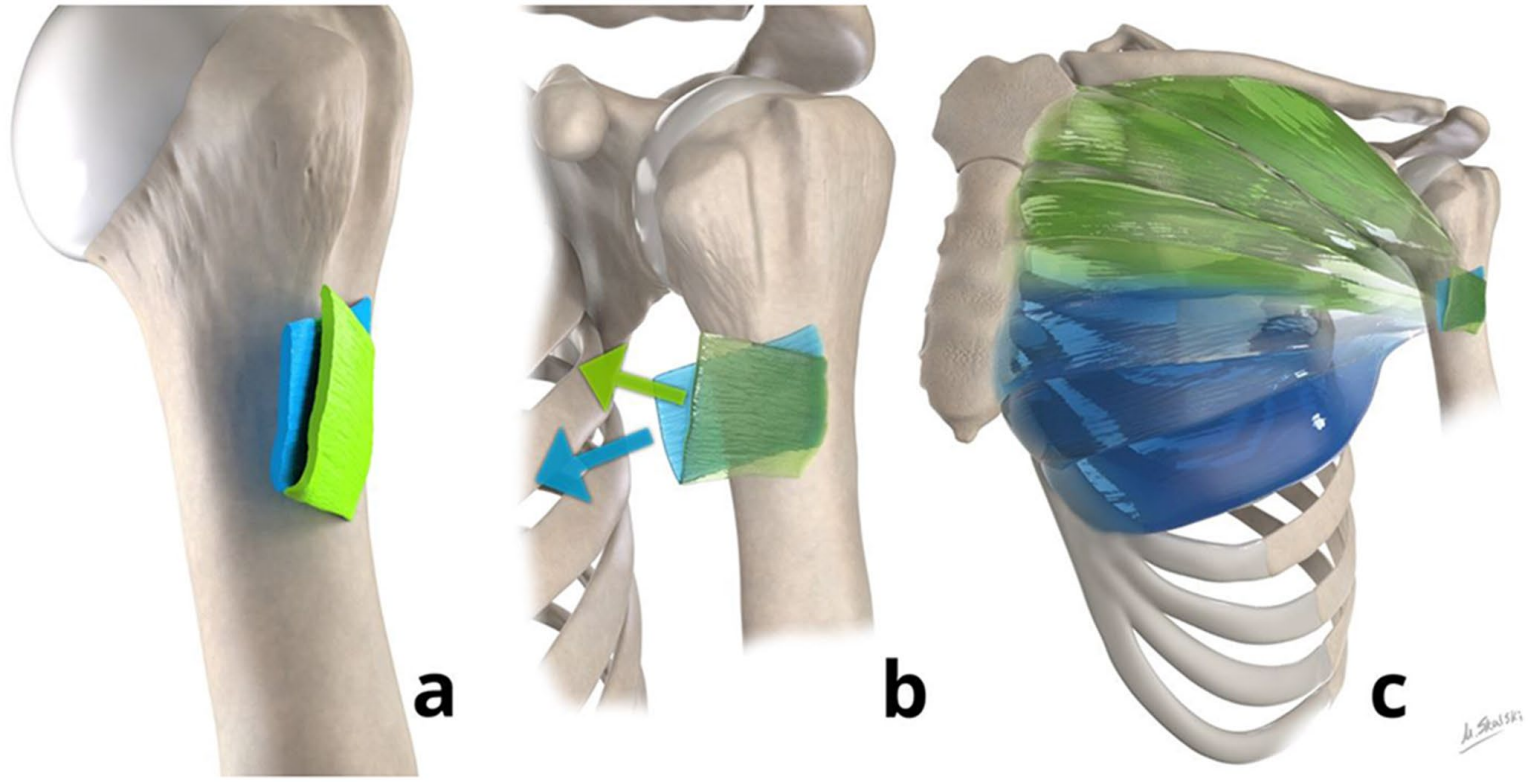
Illustrations (**a**, **b**) showing the U-shaped morphology of the anterior (green) and posterior (blue) tendon layers of the pectoralis major at the humeral insertion. The anterior layer consists of clavicular and sternal segments, while the posterior layer consists of sternal segment contribution. The orientation and contribution of the clavicular and sternal segments (**c**) to the distal tendon is also shown

This muscle consists of fibers originating from the anterior chest wall, including the clavicle and sternum, inserting onto the humerus at the lateral lip of the bicipital groove, permitting its functions of adduction, forward flexion, and internal rotation of the shoulder (Fig. 1) [9, 11, 13].

The pectoralis major muscle also contributes to stabilization of the glenohumeral joint [15, 16] and is innervated by the medial and lateral pectoral nerves. Variable distal tendon dimensions have been reported, with the width of the insertional footprint ranging between 46 and 77 mm [11].

The pectoralis major muscle consists of a clavicular head arising from the medial clavicle and a sternal (sternocostal) head which can have two to seven segments originating from the anterior manubrium, body of the sternum, first through sixth costal cartilage, and fascia of the external oblique muscle, and some authors note origination also from the fascia of the transversalis abdominal muscles [8]. Some authors subcategorize the sternal head, referring to its most inferior fibers, which arise from the fifth and sixth costal cartilage and the fascia of the abdominal muscles, as the abdominal segment of the sternal head, while others refer to this portion as a separate third, abdominal head of the pectoralis major muscle [8, 9].

### Anatomic variability

Anatomic variations of the pectoralis major have also been described and should be recognized to avoid misinterpreting such variants as pathology [7]. For example, there is variability in the overall morphology of the muscle with the clavicular head separated from the sternocostal head in about 1 in 4 cases. Rarely, the cleft is between the portion arising from the manubrium and the remainder of the sternocostal muscle rather than between the clavicular head and the sternocostal head. There is also variation in length of origin of the muscle from the clavicle and from the sternum. Fusion of the clavicular head and the deltoid muscle occurs in about 5% of cases. The number of costal cartilages from which the muscle arises is also variable, and can vary from two to seven. Typically, the muscle arises from 2nd through 6th costal cartilage (in about 60% cases) but can arise from 1st to 6th costal cartilage (about 20% cases), or from 2nd through 7th costal cartilage or from 1st through 5th costal cartilage (about 7.5% each). Variability in the length and width of the anterior and the posterior laminae, and distance of attachment from the top of the humeral head or greater tuberosity have also been described. Accessory muscles in this region including pectoralis quartus, pectoralis intermedius, pectoralis minimus, chondroepitrochlearis, and sternalis have been reported [17].

### The layered tendon

While most authors describe a bilaminar medial pectoralis major tendon, others have discussed a trilaminar appearance [1]. The widely accepted morphology of the distal (lateral) pectoralis major tendon has been described as “U-shaped” and is based on the framework of a bilaminar distal tendon attachment (Fig. 1) [2, 15]. In this model, the distal pectoralis major tendon has an anterior layer and a posterior layer, which are continuous inferiorly, creating the “U-shaped” morphology [2, 15]. While the anterior layer consists of fibers from the clavicular head and superior fibers of the sternal head, the posterior layer consists of only fibers from the sternal head [2]. Based on this “U-shaped” bilaminar distal pectoralis major tendon, a sequential order of tearing involving the tendon layers to varying degrees, ranging from partial-thickness partial-width to complete-thickness complete-width tears, has been proposed, as described later. Although this layered anatomy has been described, these layers are often fused and are not separately evident on ultrasound or MRI [15].

A multimodality investigation including histologic analysis of the distal pectoralis major tendon published in 2020 showed a unilaminar fibrocartilaginous humeral attachment on histologic assessment, suggesting that the delaminating injuries described in the literature may have arisen from a different site, and challenging the premise of the bilaminar tendon and the proposed sequence of injury [1].

### Regional anatomy

**Fig. 2:**
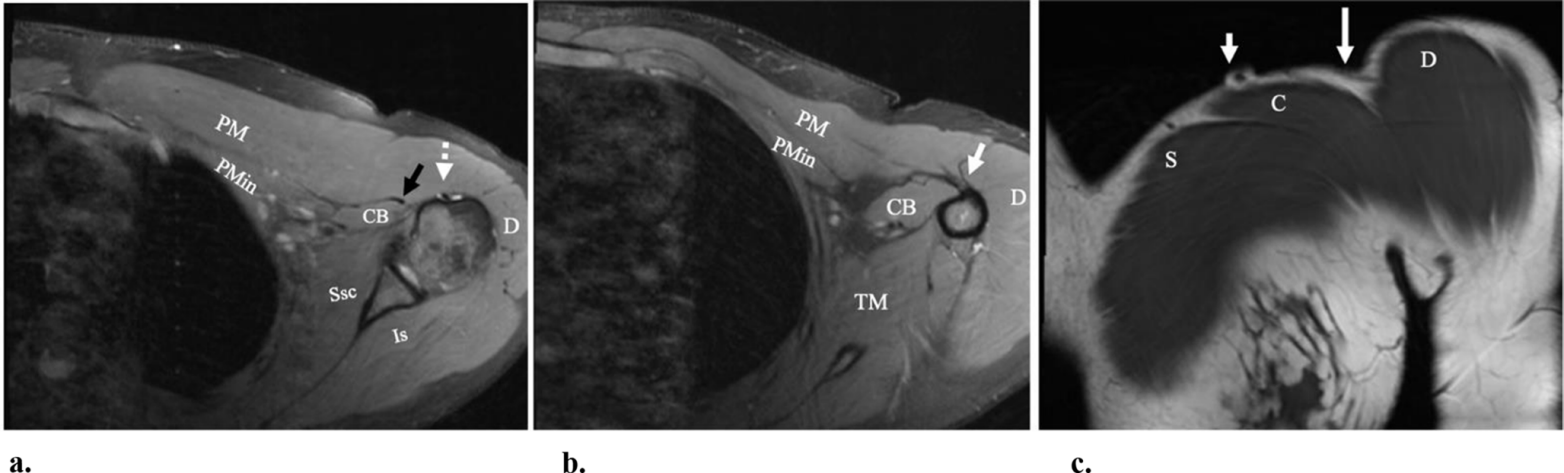
Normal anatomy in a 21-year-old-female that presented with pain in the clavicular region, with the marker (short arrow in c) overlying the site of pain. (**a**) and (**b**) Axial proton-density-weighted fat-saturated MR image shows the pectoralis major muscle (PM) at two levels, with (**a**) above the level of (**b**). More cranially in (**a**), the normal position and appearance of the long head of the biceps tendon (dashed arrow in a) in the bicipital groove is denoted. Also note the tendons of the coracobrachialis and short head of the biceps (black arrow in a). The pectoralis minor muscle (PMin), coracobrachialis muscle (CB), subscapularis muscle (Ssc), infraspinatus muscle (Is) and the deltoid muscle (**D**) are also shown. More caudally in (**b**), the PM tendon as it approaches the humeral insertion (arrow in b) at the lateral lip of the bicipital groove is shown. The teres major muscle (TM) is shown, in addition to the other abbreviated muscle from (a). (c) Coronal T1-weighted MR image shows the clavicular (**C**) and sternal (S) heads of the pectoralis major muscle. Note the deltopectoral groove (long arrow in c), which separates the deltoid muscle (**D**) from the head of the pectoralis major muscle. The obliquely oriented tubular low signal in the subcutaneous fat in this groove, subjacent to the arrow, is the cephalic vein

Lateral lip of the bicipital groove: The distal pectoralis major tendon inserts on this site, distinguishing this as a useful landmark in the assessment for pectoralis major injury **(**Fig. 2**)**. The insertion of the pectoralis major occurs at the level of the humeral insertion of the latissimus dorsi and teres major, and origin of the lateral head of the triceps muscle [7]. Non-visualization of the pectoralis major insertion between the levels of the humeral attachment of the lateral head of the triceps and the deltoid tuberosity should prompt examination for possible tear and medial retraction on MRI [18].

Long head of the biceps brachii tendon and muscle: The pectoralis major tendon inserts on the lateral lip of the bicipital groove, forming a retinaculum which maintains the long head of the biceps brachii tendon against the anterior humerus at this level (Fig. 2) [19]. The long head of the biceps brachii muscle is deep to the pectoralis major tendon as it courses to its humeral attachment. Anterior displacement of the biceps tendon by more than 4.5 mm relative the humerus is associated with complete full-thickness tears of the pectoralis major tendon [19].

Short head biceps brachii muscle: The short head biceps brachii muscle is deep to the pectoralis major tendon and medial to the long head of the biceps tendon.

Coracobrachialis muscle: This muscle is deep to the pectoralis major tendon, originates from the coracoid process of the scapula as a conjoined tendon with the short head of the biceps, and inserts onto the medial humerus.

Deltoid muscle: The deltoid muscle consists of an anterior, middle and posterior components [16]. The anterior deltoid muscle is superficial to the pectoralis major tendon. Fusion of the pectoralis major muscle with the anterior component of the deltoid muscle has been described [1, 7].

Deltopectoral groove: The deltopectoral groove refers to the interval between the pectoralis major muscle belly and the anterior head of the deltoid muscle, serving as a surgical landmark, and contains the cephalic vein [20]. Edema or fluid in this region can be associated with injury of the pectoralis major muscle [7]. On axial images, the pectoralis major myotendinous junction is deep to the deltopectoral groove [18].

Pectoralis minor muscle: This triangular muscle is deep to the pectoralis major muscle. The pectoralis minor muscle originates from the external surface of the third, fourth and fifth ribs, and sometimes also of the second and sixth ribs and inserts at the medial and superior surfaces of the coracoid process [16].

### Mechanisms of injury

Indirect trauma, particularly with the bench press maneuver, is the most common mechanism of pectoralis major injury [5, 8, 11]. Males between 20 and 40 years old represent the typical demographic with this injury [11]. In this scenario, with the humerus extended, there is eccentric muscle contraction, and the shorter, maximally stretched inferior sternal head muscle fibers are predisposed to tear [6, 8, 12]. Various types of direct trauma, including occupational injuries, have also been identified as injury mechanisms [11].

### Injury classification systems

Multiple imaging-based classification systems of pectoralis major injury have been proposed. There is no current consensus regarding the definition of acute versus chronic injuries of the pectoralis major based on the time interval since injury clinically or by imaging, and various definitions have been used [11]. There is also variability in the degree of detail across radiology and operative reports regarding these injuries. In practice, communication between the ordering clinicians, including surgeons, and radiologists regarding the specific goals of imaging such as reporting details is needed to ensure optimal patient care.

In 2012, ElMaraghy and Devereaux proposed a standardized classification system for pectoralis major injuries, which consisted of three parameters: injury acuity (distinguishing an acute injury as within a timeframe of six weeks), tear location, and tear extent [15]. The authors defined three tear location subcategories: muscle origin or belly, at or between the musculotendinous junction and tendinous insertion, and bony avulsion fracture from the humerus. The parameter of tear extent was based on the framework of a bilaminar distal tendon attachment, described as “U-shaped” [15]. Within this framework, as described above, the distal pectoralis major tendon has an anterior layer and a posterior layer, which are continuous inferiorly, creating the “U-shaped” morphology [2, 15]. Each layer measures approximately 2 millimeters in thickness [2]. According to this classification system, tear extent has two features to characterize: thickness in the anterior-posterior dimension, which can include one or both of the described two layers, and width in the proximal to distal dimension, which can be incomplete or complete [15].

Based on the “U-shaped” pectoralis major common tendon framework, a sequential order of tear progression has been proposed, first involving the posterior layer, which consists of only sternal fibers, at its most inferior components and then progressing to involve its more superior segments, followed by the clavicular head with increasing loads [15]. Although some injuries may not occur in this typical pattern and characterization can be limited by regional edema [7], this classification system proposed by ElMaraghy and Devereaux provides a useful anatomic-based approach to conceptualizing these injuries within this framework.

In this classification system, tears including only a portion of the posterior layer are defined as partial-thickness partial-width, and tears involving the entire posterior layer without involvement of the anterior layer are defined as partial-thickness full-width [7, 15]. Tears involving the entire posterior layer and a portion of the anterior layer, which includes the most superior sternal segments, are classified as full-thickness partial-width, and tears involving the entire posterior layer in addition to the entire anterior layer are classified as full-thickness, full-width injuries [7, 15]. As mentioned previously, the bilaminar appearance is most commonly not evident on ultrasound or MRI. Classification into partial-thickness tears of the tendon is often made at surgery, and an awareness of this imaging limitation is important for radiologists and clinicians.

Prior to this proposal, the Tietjen classification system had been described, without a parameter for acuity, although with distinction between contusion and partial versus complete tear, with subsequent Bak modifications to include bony avulsion and tendon substance rupture [21]. In 2020, Cordasco and colleagues proposed another classification system, which distinguished a type I injury as a contusion, type II as a tear involving one head, and type III as a tear involving multiple heads, with multiple type II and III subtypes based on the location of the tear [21].

According to a critical analysis of the literature published by Magone and colleagues in 2021, however, there is no consensus regarding the definition of acute versus chronic injuries of the pectoralis major or regarding the optimal timeline for surgical intervention with either repair or reconstruction [11]. A combination of case-based factors including the patient’s age and activity level and features of the injury contribute to the decision for non-operative versus operative management [12].

### MRI characterization of pectoralis major injuries in practice

As discussed, imaging characterization of pectoralis major injuries is limited in comparison to the detailed histological reference, and partial-thickness tears are often diagnosed intraoperatively. In light of the limitations of imaging, the authors prefer to apply a standard three-part MRI grading system, which will be described in *MRI Features of Injury and Tear Characterization*, to provide information that can be used to guide treatment. This preferred system of the standard three grade classification incorporates a fundamental element of the Tietjen and ElMaraghy systems: the distinction between a partial versus complete tear. Although the ElMaraghy classification is a more comprehensive system based on the concept of the “U- shaped” tendon, due to the limitation of MRI and ultrasound including difficulty distinguishing the two layers, in practice, the authors prefer this simpler approach of grading.

### Locations of Pectoralis Major Tears

**Fig. 3:**
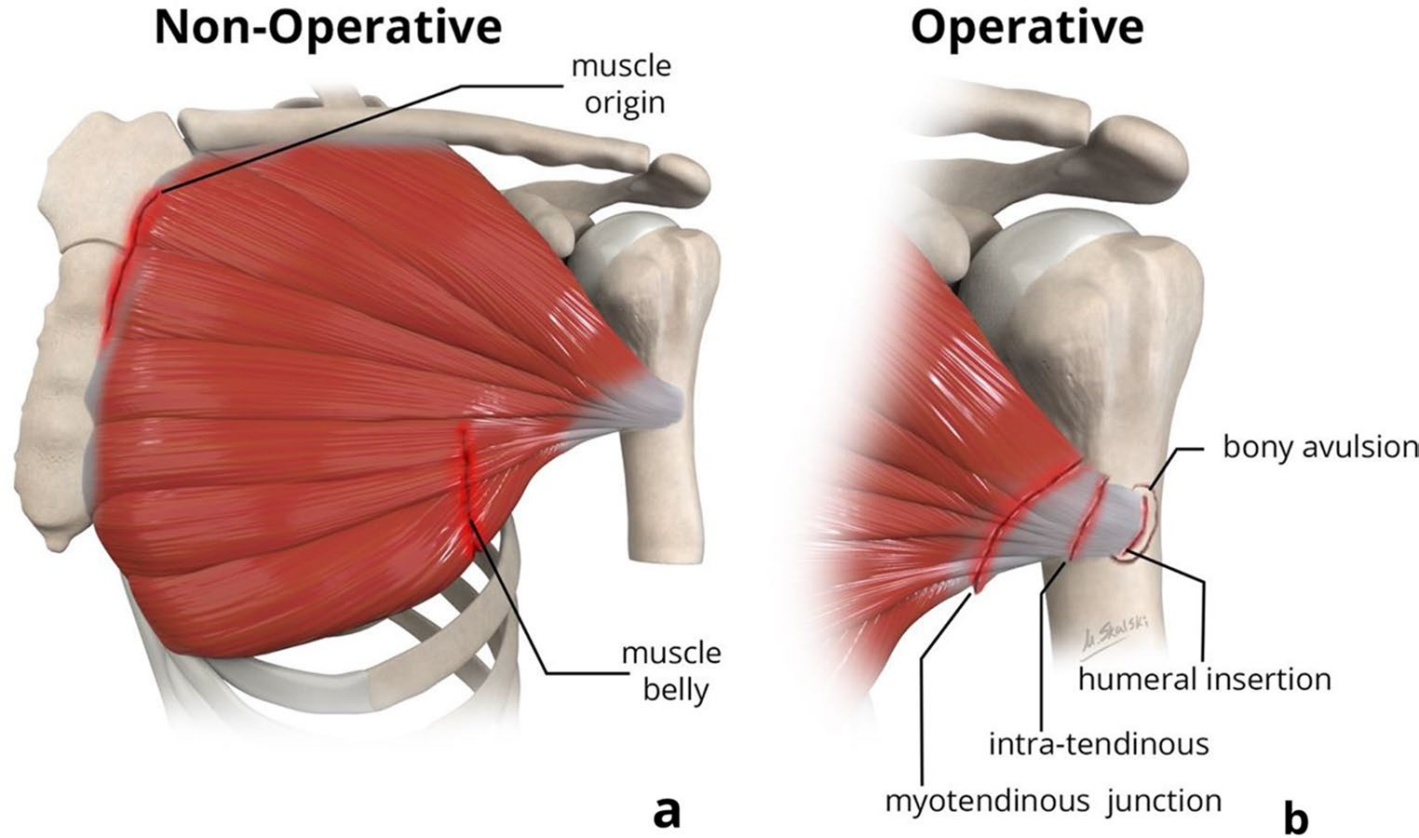
Coronal illustrations showing variable sites of pectoralis major injuries, demarcated as an irregular black line. Injuries that are typically managed non-operatively (**a**) occur at the muscle origin and at the muscle belly. In contrast, injuries that are typically managed operatively (**b**) occur at the myotendinous junction, are intra-tendinous, or are related to avulsion with or without bone fragment from the humeral insertion

The location of pectoralis major injury contributes to clinical decision-making for management [7], and can be described as involving its muscle origin, muscle belly, myotendinous junction, intra-tendinous, or the humeral insertion (Fig. 3) [15].

As noted previously, the more inferior sternal head fibers are more predisposed to tear compared to clavicular head fibers [10–13]. While clinical management is guided by multiple patient-and case-specific factors, cases of strain and tears at the muscle origin or muscle belly are generally categorized as non-operative cases, and surgical management with various operative techniques can be considered for tears at the other locations [7, 15].

Variable relative frequencies of tear locations have been reported in the literature, with tendon avulsion from the humerus or tear at the myotendinous junction as the most common site [2, 7, 11, 22]. In an analysis of pectoralis major injuries published in 2021 based on 313 cases of pectoralis major injuries with tear location described, the most common reported site of tear was soft tissue avulsion from its humeral attachment at 75.4%, with the motendinous junction as the next most frequent site at 17.6%, and with 6.1% at the muscle belly, 0.6% as osseous avulsion from the humeral attachment, and 0.3% as trans-tendinous tears [11]. The sensitivity and limitations of MRI in detecting the various sites and types of tears will be discussed in the next section.

### Sample MRI field of view for pectoralis major assessment

**Fig. 4:**
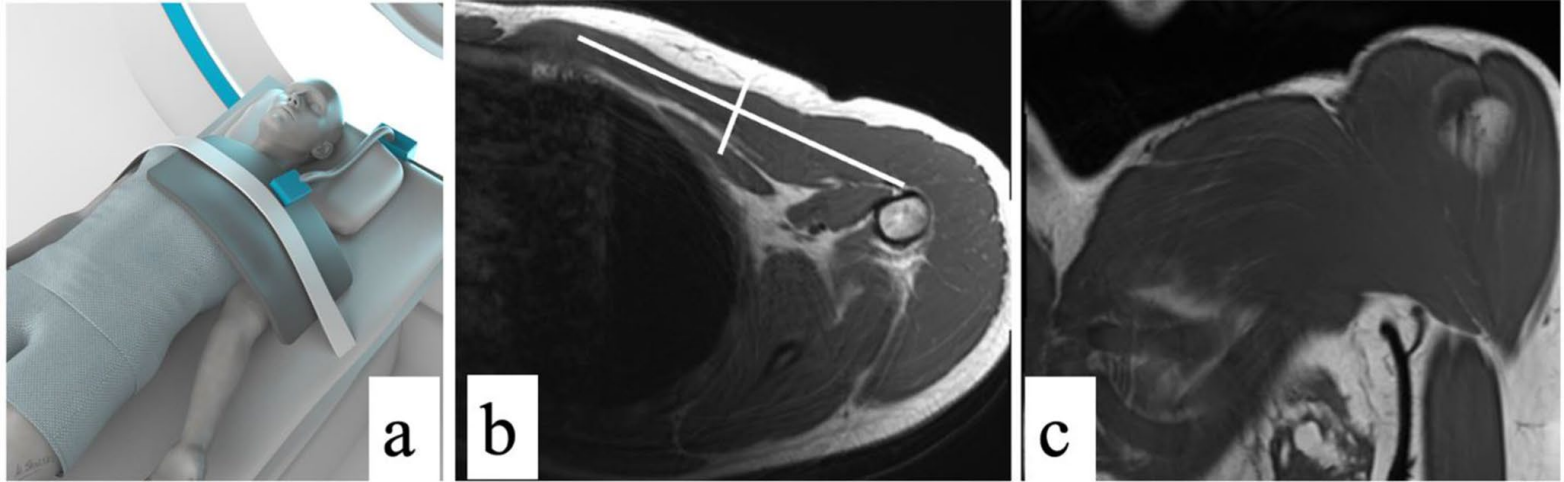
Sample supine patient positioning for MRI assessment of the pectoralis major is shown (**a**). In this example, the patient is positioned supine with a flexible coil placed over the chest on the side of interest strapped tightly to the patient to minimize respiratory artifact, and the arm is slightly abducted with external rotation to improve visibility of the tendon. The MRI field of view on a selected axial T1-weighted (**b**) and oblique coronal T1-weighted (**c**) image are shown. Reference lines for the coronal and sagittal plans are shown in (**b**). The coronal section is set parallel to the majority of the fibers of the pectoralis major muscle. The sagittal section should be set perpendicular to the coronal section

Magnetic resonance imaging protocols of the chest tailored for assessment of the pectoralis major, to include its origin and insertion, have been described, with various approaches to optimization with positioning and specific coil-type used (Fig. 4) [7, 18]. At the institutions of the authors, a body array surface coil with adequate coverage and with an overlying strap is preferred and section thickness of 3–5 mm is obtained. The advantage of the surface coil is the increased proximity of the imaged anatomy to the coil, giving a higher signal to noise ratio and thus better resolution and improved quality. When a pectoralis major injury is suspected, this specified pectoralis field of view is most useful, as a routine MRI of the shoulder will not completely include the relevant anatomy, leading to incomplete visualization of the injury [7]. Standard axial with coronal oblique and sagittal oblique planes are preferred as these planes better display this anatomy compared to standard coronal and sagittal images. Axial images on T1 non-fat suppressed and on fluid-sensitive sequences with fat-suppression often best display the pectoralis major insertion and its relationship to the regional anatomy [18]. The distal pectoralis major tendon terminates proximal to the deltoid tuberosity, which can be used as a landmark for the most caudal extent of imaging on small field of view images [18].

### MRI features of injury and tear characterization

MRI has a higher sensitivity for detecting complete tears and tendon avulsion of the pectoralis major tendon compared to partial-thickness and myotendinous junction tears, which has been partly attributed to limited delineation of the myotendinous junction on MRI [22, 23]. In a retrospective analysis published in 2016, MRI had a sensitivity of 100% in clssifying complete tears of the sternal and clavicular heads, and a sensitivity of 80% for prtial tears involving the sternal head compared to 67% for prtial tears involving the clavicular head when compared to surgical findings as the reference standard [23]. That analysis also showed a 93% sensitiviy in classifying tears of the bone-tendon junction of the sternal head compared to 80% at the mytendinous junction [23]. In the same study, there was increased MRI sensitivity in the detection of acute compared to chronic pectoralis major tears [23].

Distinguishing between tears from the humeral attachment and at the myotendinous junction on MRI can be challenging partly because a portion of the myotendinous junction is intramuscular [15], and anatomy may be obscured by regional edema in the acute setting. Additionally, the myotendinous junction of pennate muscles, such as the pectoralis major, has a more variable transition zone compared to the more discrete junction in parallel muscles [14]. It is thus useful for radiologists to understand this potential limitation in imaging and to recognize that the classification system proposed by ElMaraghy and Devereaux categorized tears “at or between the musculotendinous junction and tendinous insertion” as the same location [15].

In keeping with the type of diagnostic information obtained from routine MRI sequences, for assessment of the pectoralis major on MRI, T1-weighted images without fat-saturation are used to delineate regional anatomy while fluid-sensitive sequences, such as T2-weighted or Short Tau Inversion Recovery (STIR), elicit features of acute injury, such as edema and fluid. While specific MR imaging protocols vary based on the available MRI system and capabilities, field strengths, coils, and institutional preference, these sequences constitute the needed sequences to permit assessment of the pectoralis major.

**Fig. 5:**
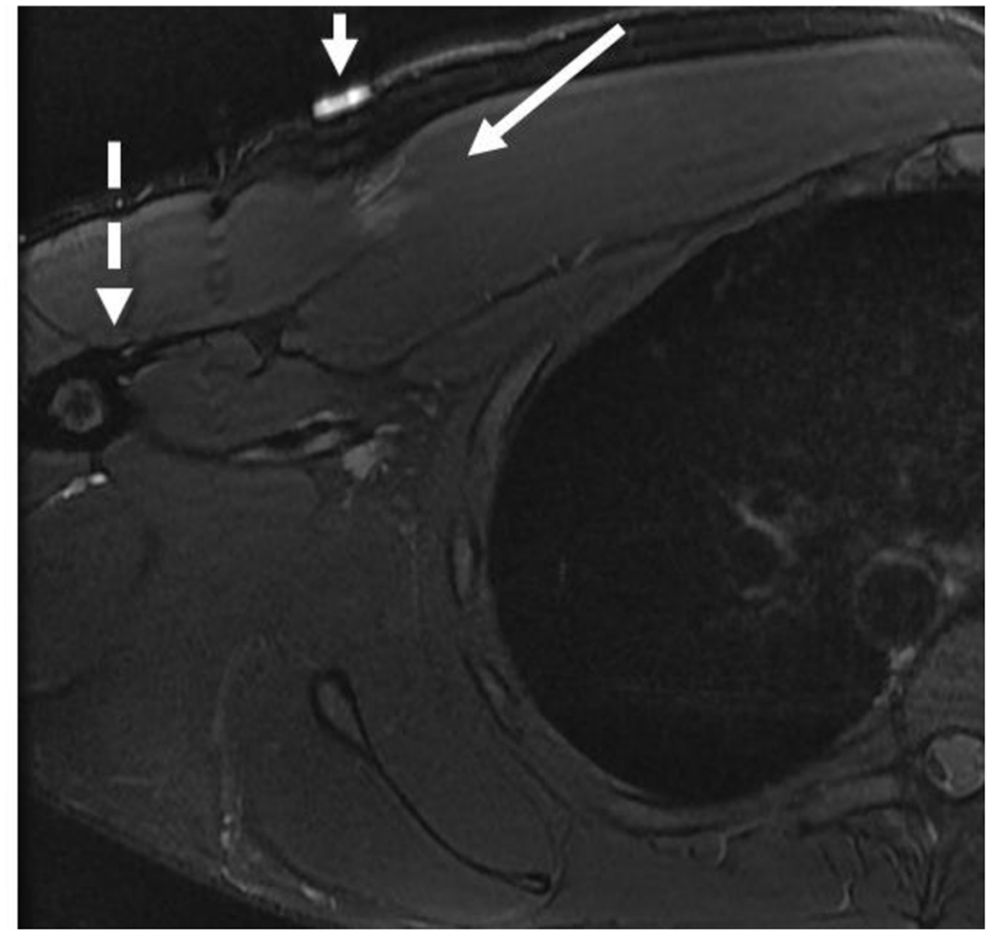
24-year-old-male with left anterolateral chest pain underlying the marker (short arrow). Axial T2-weighted with fat-saturated MR image shows feathery edema-like signal (long arrow) underlying the marker at the myotendinous junction of the sternal head of the pectoralis major muscle related to low grade (grade I) strain. Note that the distal tendon is intact (dashed arrow)

Abnormal hyperintensity on fluid-sensitive sequences is indicative of acute injury, which has a spectrum of appearances depending on the degree of injury. In a grade I injury, referring to instances of muscle strain or contusion, feathery intramuscular edema is characteristic (Fig. 5) [7].

**Fig. 6:**
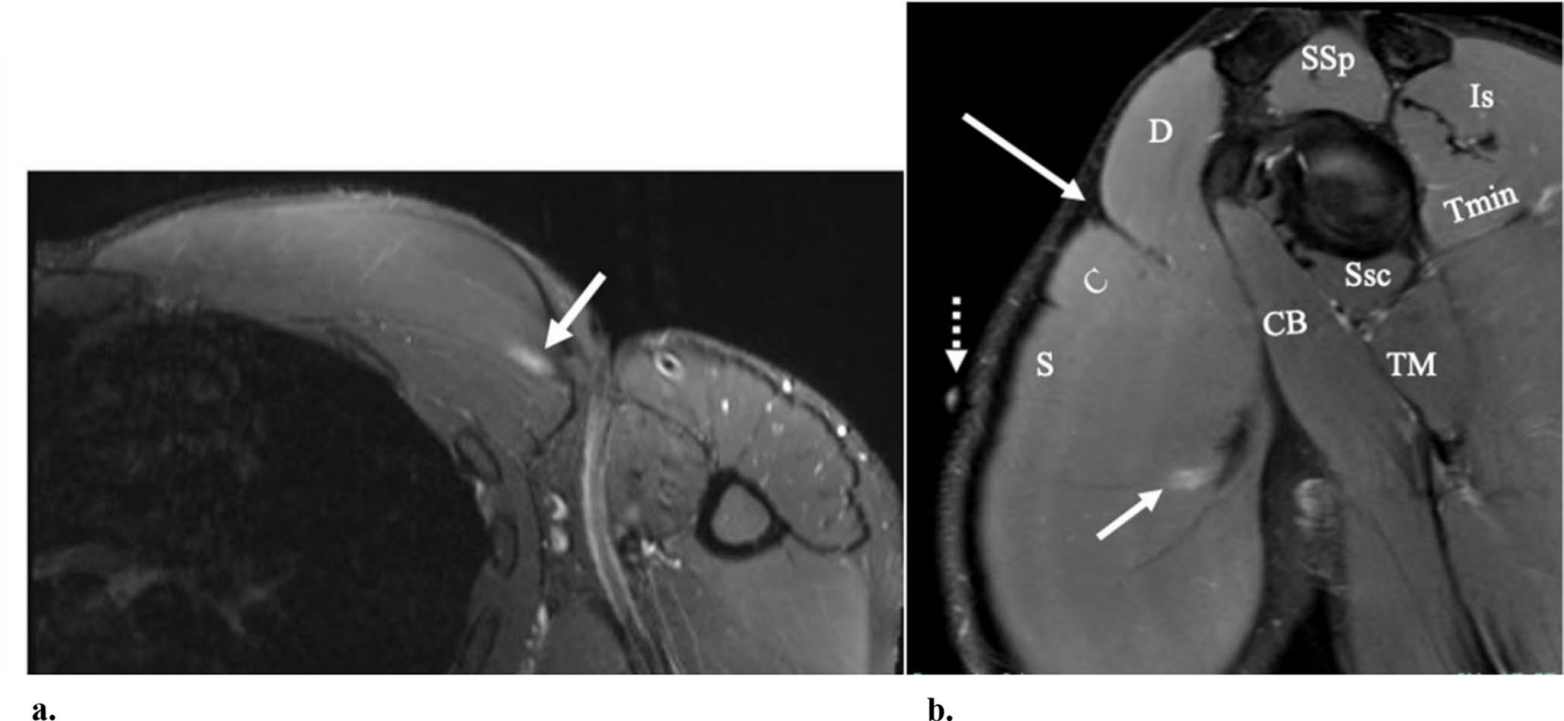
24-year-old-male with pain at the site of the external marker (dashed arrow in b). (**a**) Axial STIR MR image shows edema-like signal at the myotendinous junction of the pectoralis major (arrow), related to partial width tear at this site. (**b**) Sagittal proton-density with fat-suppression also shows the partial tear at the myotendinous junction of the sternal head (short arrow). Note the deltopectoral groove (long arrow) between the deltoid muscle (**D**) and the clavicular head (**C**) of the pectoralis major muscle. The sternal head (S) of the pectoralis major muscle is shown. The supraspinatus muscle (SSp), infraspinatus muscle (Is), teres minor muscle (Tmin), teres major muscle (TM), subscapularis muscle (Ssc), and the corachobrachialis muscle (CB) are also denoted

**Fig. 7:**
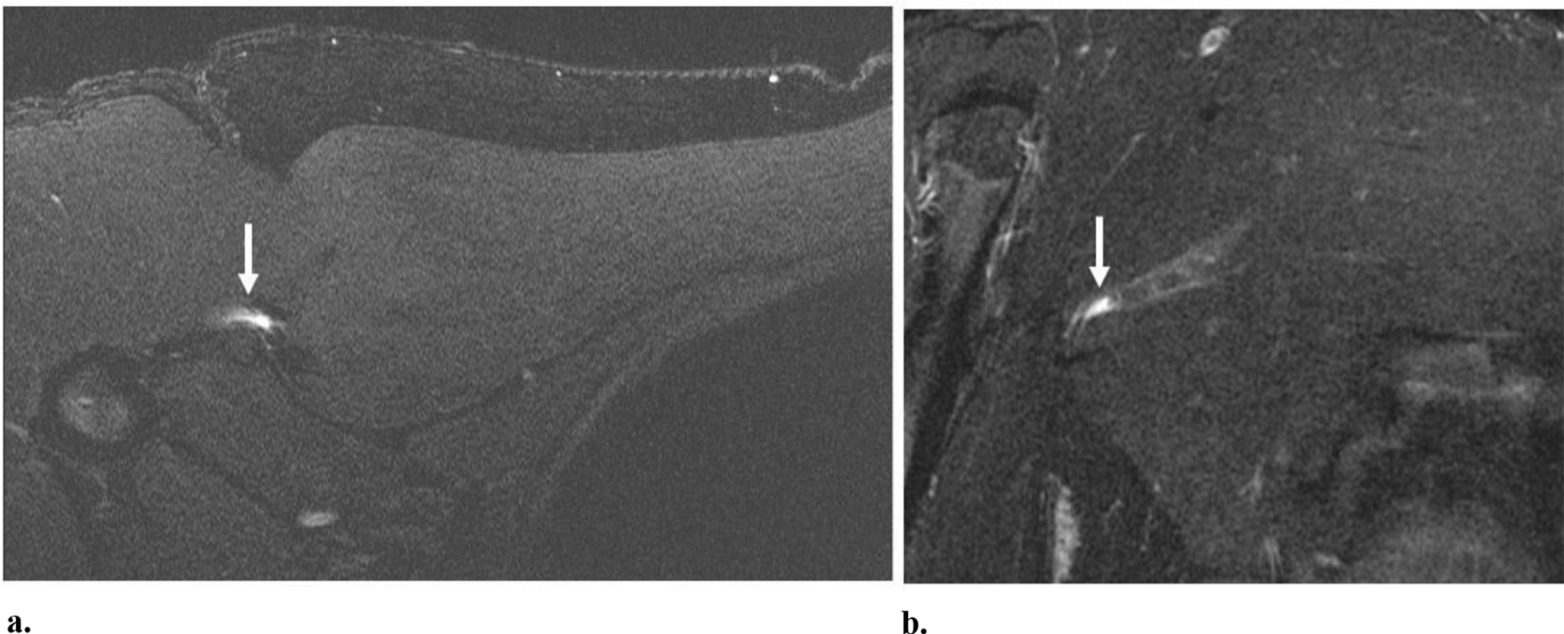
18-year-old-male that presented after pain with weightlifting. (**a**) Axial T2-weighted-fat-saturated and (**b**) coronal oblique STIR- weighted MR images show edema-like signal along the myotendinous junction (arrow) of the sternal head of the pectoralis major, representing partial-width tear

**Fig. 8:**
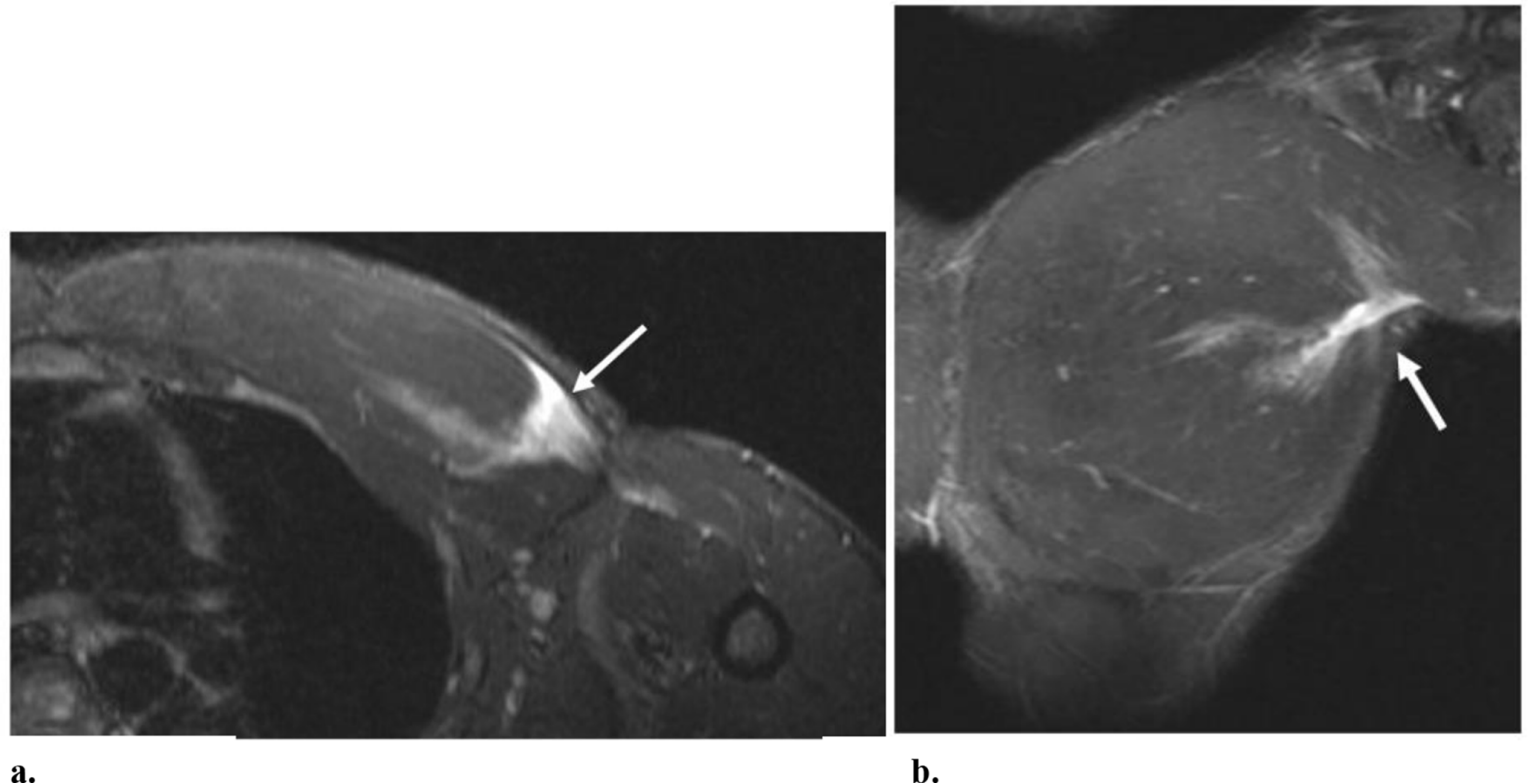
20-year-old-male football player that presented after feeling a pop in the anterior chest while bench-pressing. (**a**) Axial and (**b**) Coronal STIR MR images show a full-thickness tear on the axial image, characterized by a fluid gap, at the myotendinous junction (arrow), involving the sternal head of the pectoralis major muscle. At surgery, there was a tear at the myotendinous junction with tendinous fibers still attached to the humerus

**Fig. 9:**
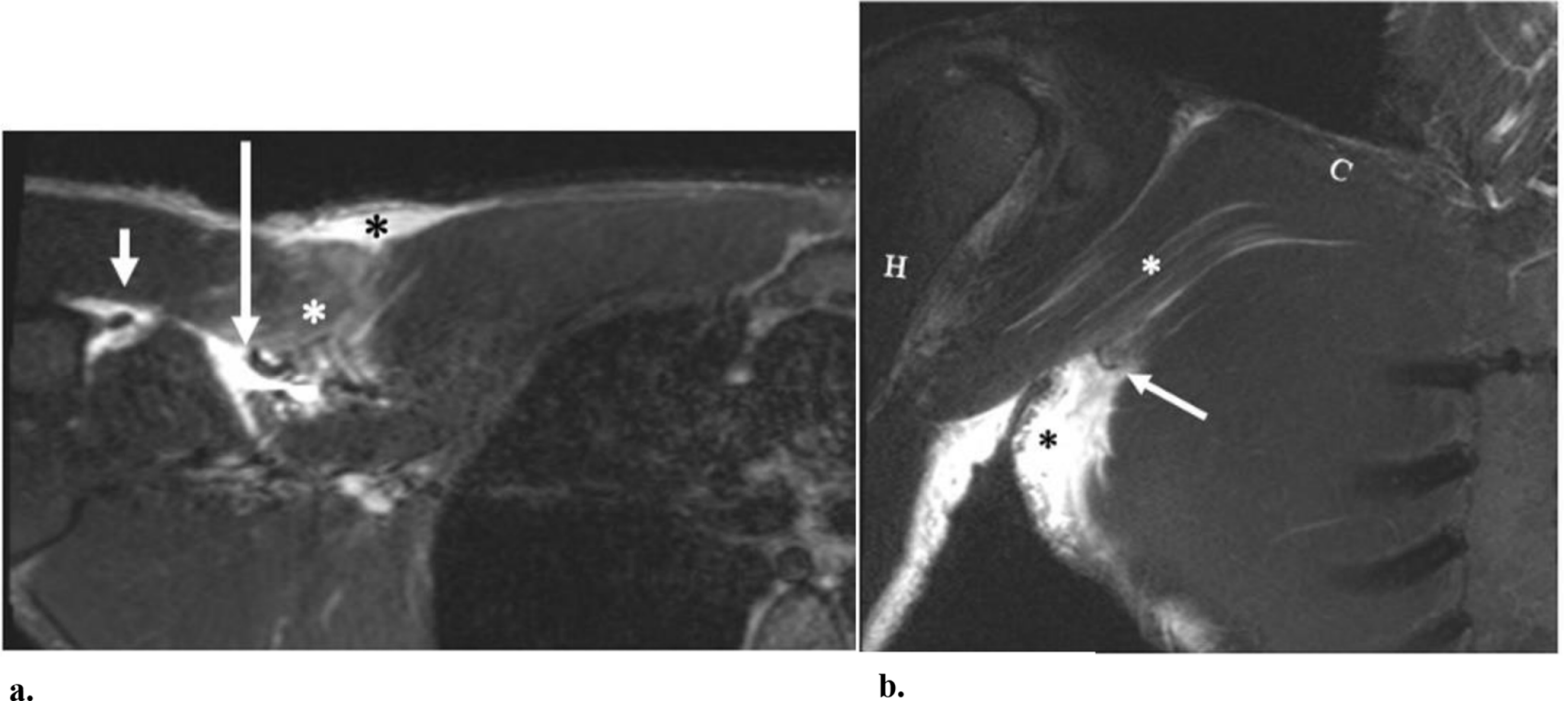
42-year-old-male that presented after feeling a pop in the anterior chest when bench-pressing. (**a**) Axial STIR MR image shows a full-thickness, partial-width tear with a medially retracted tendon stump (long arrow) originating from the sternal head of the pectoralis major with areas of intramuscular edema (white asterisk) related to sites of interstitial tears. A triangular peri-bicipital hematoma (short arrow) is shown lateral to the retracted tendon stump. Anterior subcutaneous edema is also present (black asterisk). (**b**) Coronal STIR MR image also demonstrates the retracted tendon stump (arrow) with regional edema (black asterisk). Intramuscular edema is present along the intact clavicular fibers of the pectoralis major (white asterisk). The shafts of the clavicle (**C**) and humerus (H) are also denoted. At surgery, there was a complete tear of the sternal head from its humeral attachment with medial retraction and extension of the tendon rupture into the myotendinous junction and muscle belly

A grade II injury refers to a partial tear and a grade III injury refers to a complete tear [7], each with a variable degree of muscle fiber distortion and regional edema (Figs. 6, 7, 8 and 9 [2].

In the acute setting, regional edema can obscure features of the injury [2, 7] and may limit delineation of a retracted tendon. As regional edema is nonspecific, processes such as calcific tendinitis in this region may also be diagnostic considerations, and correlations with radiographs and with the clinical history is needed. Additionally, in the setting of calcific tendinitis, the foci of mineralization would have low signal on T1 and T2 weighted sequences, as well as on T2* sequences if included if there is suspicion for this entity. The absence of tendon fibers at the humeral attachment is indicative of a tendon tear. In cases of tendon avulsion, periosteal stripping, characterized by edema superficial to the humeral cortex [24] near the tendon’s insertion, can be seen. Plain radiographs or non-contrast CT may show such bony avulsion to better advantage compared to MRI. Radiologists’ attention to concurrent imaging studies of this region for avulsion injury is important and correlation with radiographs or non-contrast CT may be considered in instances where this type of injury is suspected.

**Fig. 10:**
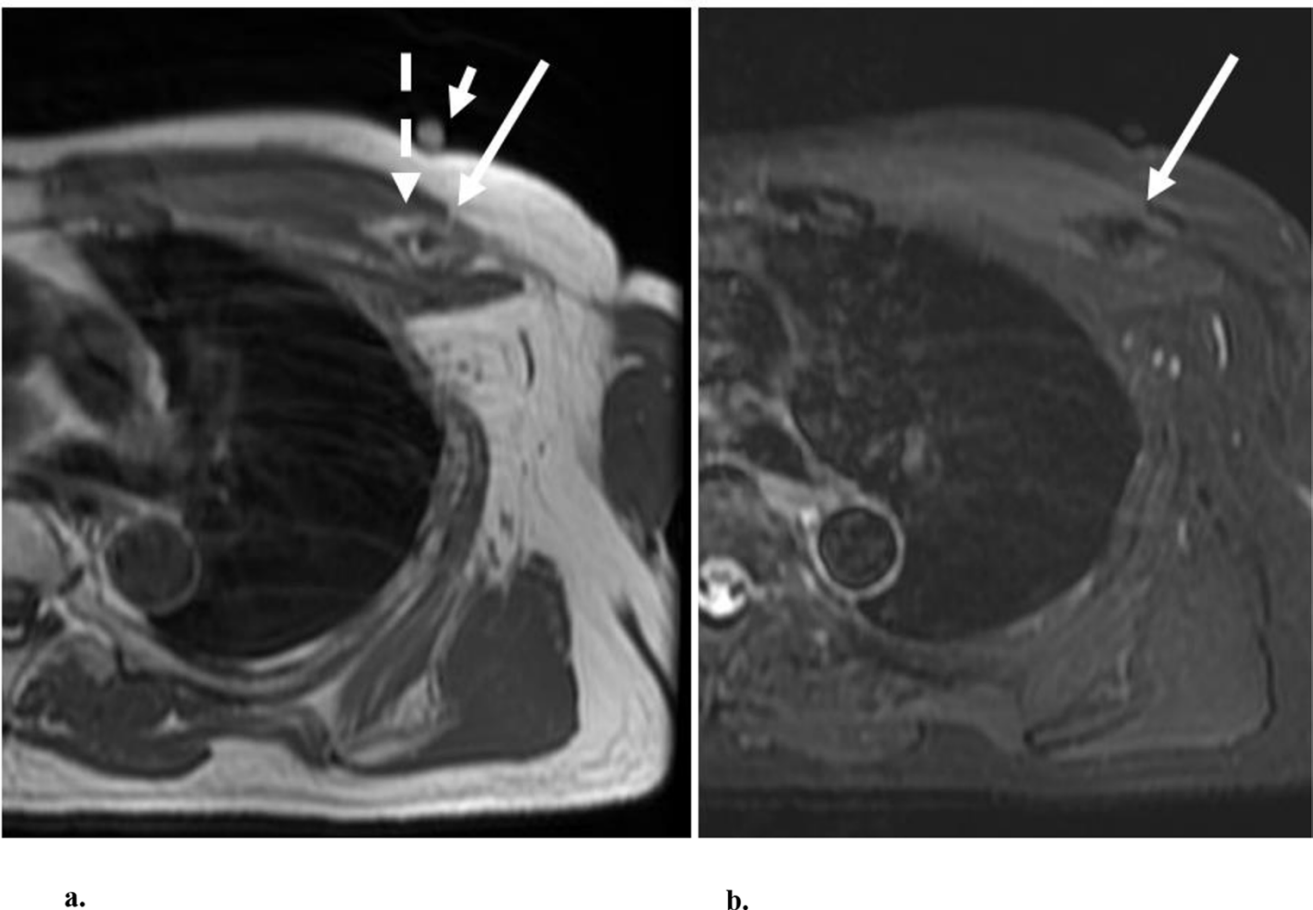
45-year-old-male that presented with left anterolateral chest pain underlying the marker (short arrow in **a**) approximately 8 months after injury of the left pectoralis major from assault. (**a**) Axial T1-weighted MR image shows nodular signal, which is relatively hypointense to muscle, in the region of the left pectoralis major myotendinous junction (long arrow). Note surrounding fat signal (dashed arrow). (**b**) Axial STIR-weighted MR image shows the corresponding low signal at this site (arrow) and the absence of edema. At surgery, there was scar tissue and a complete tear at the myotendinous junction of the sternal head (full-width partial-thickness) of the pectoralis major

In the setting of more chronic injury, distortion of the tendon fibers in the absence of edema is expected (Fig. 10). Additionally, fatty atrophy of the muscle and regional scar tissue, which is relatively low in signal on T1 and T2-weighted images, can be seen [11, 14, 24].

### Ancillary findings

**Fig. 11:**
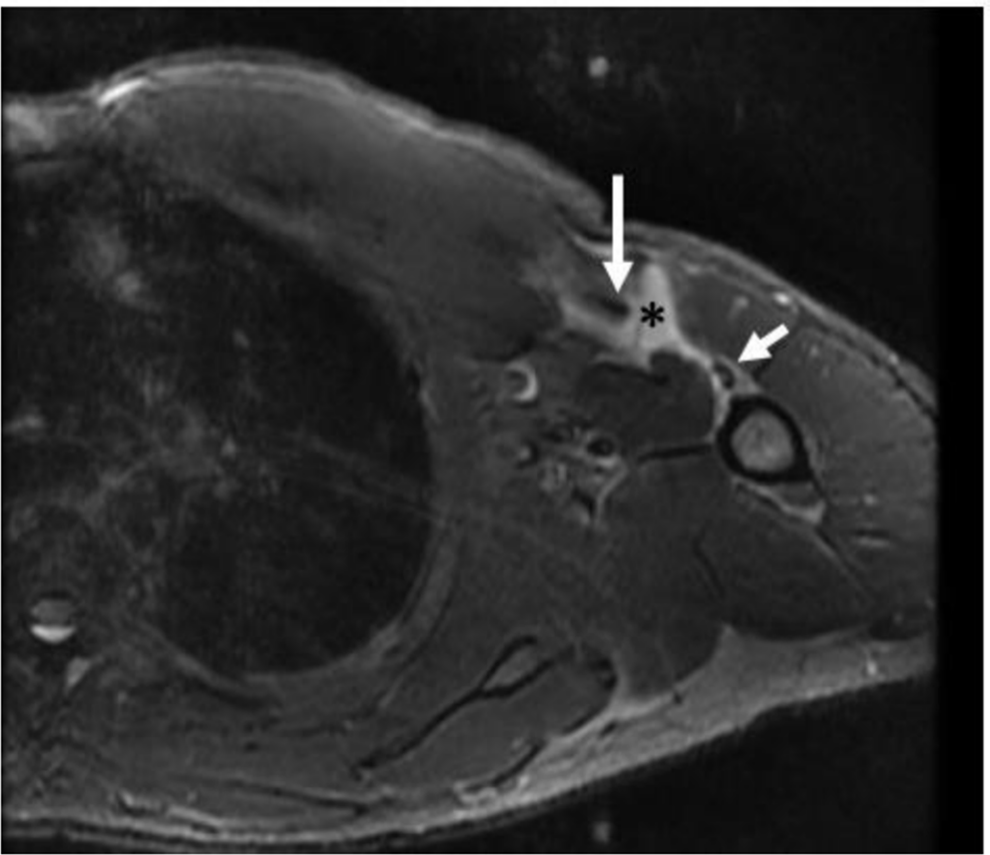
24-year-old-male that presented after feeling a painful pulling and popping sensation while bench-pressing. Axial inversion recovery MR image shows avulsion type complete (full thickness full-width) tear of the pectoralis major tendon from its humeral attachment. The avulsed tendon (long arrow) is medially retracted and there is a small amount of fluid (asterisk) laterally. Note mild anterior displacement of the long head of the biceps tendon. (short arrow). At surgery, there was a complete tear of the tendon from its humeral attachment

Anterior displacement of the biceps tendon and peri-bicipital hematoma are useful secondary findings associated with full-thickness complete tears at the humeral attachment of the pectoralis major (Fig. 11) [19]. These ancillary findings may also be evident on routine MRI studies of the shoulder, and should prompt radiologist suspicion of an incompletely included pectoralis major injury. Edema or fluid in the deltopectoral groove may also be included on MRI of the shoulder and may be associated with pectoralis major injury.

In a retrospective study published in 2018, Godoy and colleagues reported that biceps tendon displacement by more than 4.5 mm on MRI had an 86% sensitivity and 75% specificity for complete full-thickness tears with retraction from the humeral insertion in comparison to operative reports [19]. This anterior displacement of the biceps tendon was measured as the distance between the posterior contour of the biceps tendon and the anterior humeral cortex at a standardized level 6.0 cm distal to the top of the humeral head [19].

In the same study, a peri-bicipital hematoma was present on MRI in 93% of the subjects with complete full-thickness tear at surgery [19]. Each of these findings is best displayed on axial fluid-sensitive images, with a peri-bicipital hematoma characterized as T2 hyperintensity, defined by the authors of that study, as contacting more than 180 degrees of the biceps tendon in cross-Sect. [19]. While fluid associated with the biceps tendon sheath was described as having more rounded margins and coursing with the contour of the biceps tendon in its groove, peri-bicipital hematoma was distinguished by triangular morphology with its apex medially and base along the expected humeral insertion of the pectoralis major tendon [19].

### Treatment considerations

A combination of factors including the patient’s age and activity level and features of the injury such as muscle atrophy, tendon retraction, tendon quality and the degree of scarring [25], contribute to the decision for non-operative versus operative management [12].

As discussed, according to a critical analysis of the literature published by Magone and colleagues in 2021, there is no consensus regarding the chronological definition between acute versus chronic pectoralis major tears, or regarding the optimal timeline or type of surgical intervention [11]. Based on location, tears at the muscle origin or muscle belly are generally categorized as non-operative cases, and surgical management with various operative techniques can be considered for tears at the other locations [7, 15]. Non-operative management has been associated with weakness in adduction although with fair to good functional outcomes [11, 12]. In contrast, surgical intervention is associated with good to excellent functional outcomes, and is typically considered in young, active patients [11, 12].

### Future prospects

An MRI classification system of pectoralis major injuries accounting for various primary and ancillary findings, as discussed and including location, tendon retraction, muscle atrophy, and the degree of scarring, to complement clinical assessment criteria could be a valuable future direction of MR imaging interpretation and would emphasize the significance of interdisciplinary collaboration in guiding patient treatment. The establishment of a multidisciplinary task force comprising sports medicine specialists, orthopedic surgeons, and radiologists would be instrumental in the development of this multifactorial, integrated classification system. After this has been developed, retrospective analysis could be done to determine the relative and combined prognostic value of these criteria. Additionally, effort towards a consensus and standardized terminology for such injuries within musculoskeletal radiology would support clarity, inter-reader consistency, and would foster clear intra and interdisciplinary communication.

## Conclusion

An understanding of the complex anatomy and the resultant patterns of pectoralis major injuries on MRI enables radiologists to provide valuable information that contributes to the development of the patient-centered treatment plan in cases of injury. Many patients after this injury will present to the emergency department and it is important for emergency radiologists to understand the imaging findings of these injuries. Diagnosis with MRI can avoid surgical delay and improve patient outcomes [3]. This article highlights the background of the classification system of such injuries, and the essential and ancillary findings on MRI of pectoralis major injuries.

## Data Availability

Not applicable.
